# A machine learning framework for predicting drug–drug interactions

**DOI:** 10.1038/s41598-021-97193-8

**Published:** 2021-09-02

**Authors:** Suyu Mei, Kun Zhang

**Affiliations:** 1 Software College, Shenyang Normal University, Shenyang, 110034 China; 2 Bioinformatics Core of Xavier RCMI Center for Cancer Research, Department of Computer Science, Xavier University of Louisiana, New Orleans, LA 70125 USA

**Keywords:** Biochemistry, Biological techniques, Biotechnology, Computational biology and bioinformatics, Drug discovery, Systems biology

## Abstract

Understanding drug–drug interactions is an essential step to reduce the risk of adverse drug events before clinical drug co-prescription. Existing methods, commonly integrating heterogeneous data to increase model performance, often suffer from a high model complexity, As such, how to elucidate the molecular mechanisms underlying drug–drug interactions while preserving rational biological interpretability is a challenging task in computational modeling for drug discovery. In this study, we attempt to investigate drug–drug interactions via the associations between genes that two drugs target. For this purpose, we propose a simple f drug target profile representation to depict drugs and drug pairs, from which an l_2_-regularized logistic regression model is built to predict drug–drug interactions. Furthermore, we define several statistical metrics in the context of human protein–protein interaction networks and signaling pathways to measure the interaction intensity, interaction efficacy and action range between two drugs. Large-scale empirical studies including both cross validation and independent test show that the proposed drug target profiles-based machine learning framework outperforms existing data integration-based methods. The proposed statistical metrics show that two drugs easily interact in the cases that they target common genes; or their target genes connect via short paths in protein–protein interaction networks; or their target genes are located at signaling pathways that have cross-talks. The unravelled mechanisms could provide biological insights into potential adverse drug reactions of co-prescribed drugs.

## Introduction

Drug–drug interactions (DDIs) have been recognized as a major cause of adverse drug reactions (ADRs) that leads to rising healthcare costs^1^. Antagonistic drug–drug interactions may occur when a patient takes more than one drug concurrently and potentially result in adverse side effects and toxicities^2^. In many cases, drug–drug interactions are hardly detected during the clinical trial phase, and arbitrary co-prescription of drugs without prior knowledge potentially poses serious threats to patient health and life^3^. Cytochrome-P450 (CYP450) isoforms (e.g., CYP1A2, CYP2C8, CYP2C9, CYP2C19, CYP2D6 and CYP3A4/5) take the responsibility to metabolize the majority of available drugs and frequently cause antagonistic drug–drug interactions^4^. For instance, CYP1A2 metabolizes both drug Theophylline and Duloxetine. If the stronger substrate Duloxetine competes with the weaker substrate Theophylline to bind to the active site of CYP1A2, breakdown of Theophylline will be reduced, leading to increased plasma levels of theophylline and potential side-effects like headache, nausea and vomiting^5^. To reduce the risk of potential adverse drug reactions, it is crucial to examine in advance whether co-prescribed drugs interact. Drug–drug interactions could be identified via in vitro or in vivo experiments as well as in silico computational methods. However, the former two approaches are very costly and in some cases are impossible to be carried out because the serious side effects DDIs elicited in experiments could do irreversible damages to human health^6^. With the advancement of pharmacogenomics, recent years have witnessed much effort to develop data-driven in silico computational methods to predict drug–drug interactions and their efficacy, although the “black-box” machine learning and artificial intelligence models sometimes frustrates the experimental pharmacologists in terms of multidisciplinary gap and practical successes^7^

As regards drug–drug interactions, existing computational methods could be roughly classified into three categories, namely similarity-based methods^8–11^, networks-based methods^12–16^ and machine learning methods^17–25^. Similarity-based methods directly infer drug–drug interactions on the basis of similarity scores between drug profiles. Vilar et al.^8^ have reviewed several drug profiles, such as pharmaceutical profiles, gene expression profiles and phenome profiles, which have been used to infer drug repurposing, drug adverse effects and drug–drug interactions. Among these profiles, drug structural profiles could be well interpreted based on the assumption that structurally similar drugs tend to target the same or functionally-associated genes to produce similar drug efficacies^9^. The other major concern of similarity-based methods is to develop effective metrics to measure similarity between drug profiles. Ferdousi et al.^10^ choose the optimum measure from a dozen of similarity metrics between drug target profiles (e.g., inner product, Jaccard similarity, Russell-Rao similarity and Tanimoto coefficient) to infer DDIs. In spite of simple and intuitive interpretation, similarity-based methods are easily affected by noise, for instance, the thresholding of similarity scores is seriously affected by false DDIs.

The second category of methods, i.e., networks-based methods, could be further classified into drug similarity networks-based methods^12–14^ and protein–protein interaction (PPI) networks-based methods^15,16^. Drug similarity networks-based methods s predict novel links/DDIs via networks inference on the drug–drug similarity networks constructed via a variety of drug similarity metrics, e.g., matrix factorization^12,13^, block coordinate descent optimization^14^. Similar to the similarity-based methods^8–11^, these methods also resort to the similarities between drug structural profiles to infer DDIs. Comparatively, networks-based methods are more robust against noise than direct similarity-based methods. However, drug–drug interactions do not mean direct reactions between two structurally-similar drug molecules but synergistic enhancement or antagonistic attenuation of each other’s efficacy. When two drugs take actions on the same genes, associated metabolites or cross-talk signaling pathways, the biological events that two co-prescribed drugs influence or alter each other’s therapeutic effects may very well happen^10^. In this sense, the knowledge about what two drugs target is more useful and interpretable than drug structural similarity to infer drug–drug interactions, especially for the potential interactions between two drugs that are not structurally similar.

The PPI networks-based methods^15,16^ assume that two drugs would produce unexpected perturbations to each other’s therapeutic efficacy if they simultaneously act on the same or associated genes, so that these methods have the merit of capturing the underlying mechanism of drug–drug interactions. Park et al.^15^ assume two drugs interact if they cause close perturbation within the same pathway or distant perturbation within two cross-talk pathways, wherein the distant perturbation is captured via random walk algorithm on PPI networks. Huang et al.^16^ also consider drug actions in the context of PPI networks. In their method, the target genes together with their neighbouring genes in PPI networks are defined as the target-centred system for a drug, and then a metric called S-score is proposed to measure the similarity between two drugs’ target-centered systems to infer drug–drug interactions. To date, PPI networks are far from complete and contain a certain level of noise so as to be restricted in the application to inferring drug–drug interactions.

The third category of methods, i.e., machine learning methods, has been widely used to infer drug–drug interactions^17–25^. Most of these methods focus on improving the performance of drug–drug interactions prediction via data integration. In these methods, data integration attempts to capture multiple aspects of information of a single data source or combining multiple heterogeneous data sources. Dhami et al.^17^ attempt to combine multiple similarity metrics (e.g., molecular feature similarity, string similarity, molecular fingerprint similarity, molecular access system) from the sole data of drug SMILES representation. The other methods^18–25^ all combine multiple data sources. Data integration often combines diverse feature information such as drug adverse drug reactions (ADR)^18–20,23,24^, target similarity^18–20,22–24^, PPI networks^23,24^, signaling pathways^19^ and so on. Among these features, the information of drug chemical structures in the form of SMILES descriptors is most frequently used^17–24^. The machine learning frameworks used to integrate heterogeneous data include ensemble learning^18,19^, kernel methods^17,20^ and deep learning^21,22^. Empirical studies show that data integration surely enrich the description of drugs from multiple aspects and accordingly improves the performance of drug–drug interaction prediction. However, data integration suffers from two major drawbacks. One drawback is that data integration increases data complexity. In most cases, we do not know which information is the most important and indispensable for predicting drug–drug interactions. Some information may contribute less to the prediction task. More importantly, data integration renders data constraint more demanding. Once any aspect of feature information is not available, e.g., drug molecular structure, the trained model may fail to work. Actually, single-task learning without data integration also can achieve satisfactory predictive performance, e.g., deep learning on available DDI networks only^25^. The other drawback of data integration is that the molecular mechanisms underlying drug–drug interactions is often ignored or drowned by the information flood. As results, the model is trained like a black-box and the predictions are hard to interpret in biological sense. Recent studies have revealed some molecular mechanisms drug–drug interactions, e.g., targeted gene profile and signaling pathway profile^26^. This information needs to be considered to increase model interpretability.

In this study, we attempt to simplify the computational modeling for drug–drug interaction prediction on the basis of potential drug perturbations on associated genes and signaling pathways. We assume that two drugs potentially interact when a drug alters the other drug’s therapeutic effects through targeted genes or signaling pathways. For this sake, only the known target genes of drugs taken from DrugBank^27^ are used to train a predictive model without the information of drug structures or adverse drug reactions that are hard to represent and potentially are not available. The drug target profile is actually a binary vector indicating the presence or absence of a gene and the target profiles of two drugs are simply combined into a feature vector to depict a drug pair. To counteract the potential impact of noise, we choose l_2_-regularized logistic regression as the base learner. The proposed framework is evaluated via cross validation and independent test, wherein the external test data are taken from the comprehensive database^28^. We further propose several statistical metrics based on protein–protein interaction networks and signaling pathways to measure the intensity that drugs act on each other.

## Data and methods

### Data

The known drug–drug interactions and drug–gene interactions are extracted from DrugBank^27^. As we use drug target profile to represent drugs and drug pairs, only the drugs that have been discovered to target at least one human gene are studied in this work. As results, we totally extract 6066 drugs and 2940 targeted human genes from DrugBank^27^. There are 915,413 drug–drug interactions and 23,169 drug–gene interactions associated with these drugs. As drug–drug interaction prediction is essentially a problem of binary supervised learning, we use the 915,413 drug pairs as the positive training data and randomly sample another 915,413 drug pairs from the 6066 drugs as the negative training data. The two classes of data are ensured to have no overlap.

The comprehensive database^28^ provides a large repository for drug–drug interactions from experiments and text mining, some of which come from scattered databases such as DrugBank^27^, KEGG^29^, OSCAR^30^ (https://oscar-emr.com/), VA NDF-RT^31^ and so on. After removing the drug–drug interactions that already exist in DrugBank^27^, we totally obtain 13 external datasets as positive independent test data, for instance, the largest 8188 drug–drug interactions from KEGG^29^. To estimate the risk of model bias, we randomly sample 8188 drug pairs as negative independent test data. These drug pairs are not overlapped with the training data and the positive independent test data.

To quantitatively estimate the intensity that two drugs perturbate each other’s efficacy, we build up comprehensive physical protein–protein interaction (PPI) networks from existing databases (HPRD^32^, BioGRID^33^, IntAct^34^, HitPredict^35^. We totally obtain 171,249 physical PPIs. From NetPath^36^, we obtain 27 immune signaling pathways with IL1–IL11 merged into one pathway for simplicity. From Reactome^37^, we obtain 1846 human signaling pathways.

### Drug target profile-based feature construction

Drugs act on their target genes to produce desirable therapeutic efficacies. In most cases, drug perturbations could disperse to other genes through PPI networks or signaling pathways, so as to accidentally yield synergy or antagonism to the drugs targeting the indirectly affected genes. In this study, we depict drugs and drug pairs using drug target profile only. For each drug ${d}_{i}$ in the DDI-associated drug set $D$, its targeted human gene set is denoted as ${G}_{{d}_{i}}$. The entire target gene set is defined as follows.

1 $$G={\cup }_{{d}_{i}\in D}{ G}_{{d}_{i}}$$

For each drug ${d}_{i}$, drug target profile is formally defined as follows.

2 $$V_{{d_{i} }} \left[ g \right] = \left\{ {\begin{array}{*{20}l} {1,} \hfill & {g \in G_{{d_{i} }} \Lambda g \in G} \hfill \\ {0,} \hfill & {g \notin G_{{d_{i} }} \Lambda g \in G} \hfill \\ \end{array} } \right.$$

Then the drug target profile of a drug pair (${d}_{i},{d}_{j}$) is defined by combining the target profile of ${d}_{i}$ and ${d}_{j}$ as follows.

3 $${V}_{{(d}_{i},{d}_{j})}\left[g\right]={V}_{{d}_{i}}\left[g\right]+{V}_{{d}_{j}}\left[g\right], g\in G$$

The genes $g\notin G$ are discarded. The simple feature representation of drug target profile intuitively reveals the co-occurrence patterns of genes that a drug or drug pair targets. As an intuitive example, assuming the entire gene set $G=\{TF,ALB,XDH,ORM1,ORM2\}$, drug Patisiran (DB14582) targets the genes {ALB, ORM1, ORM2} and drug Bismuth Subsalicylate (DB01294) targets the genes {ALB, TF}, then Patisiran is represented with the vector [0, 1, 0, 1, 1] and Bismuth Subsalicylate is represented with the vector [1, 1, 0, 0, 0]. The drug pair (Patisiran, Bismuth Subsalicylate) is represented with the combined vector [1, 2, 0, 1, 1], which is used as the input of the base learner. All the data including the training set and the test set have the same feature descriptors. It is noted that all the target genes are chosen to represent drugs and drug pairs without giving priority or importance to the features, because the known target genes are very sparse and many target genes are unknown. If feature selection with importance weights is conducted, many drugs and drug pairs would be represented with null vector.

### L_2_-regularized logistic regression as base learner

L_2_-regularized logistic regression^38^, well-known for its fast fitting large training data and penalizing potential noise and overtraining, is adopted as the base learner in this study. Given the training data *x* and labels *y* with each instance ${x}_{i}$ corresponding a class label ${y}_{i}$, i.e., $({x}_{i},{y}_{i}),i=\mathrm{1,2},...,l;{x}_{i}\in {R}^{n};{y}_{i}\in \{-1,+1\}$, the decision function of logistic regression is defined as $f(x)=\frac{1}{1+\mathit{exp}(-y{\omega }^{T}x)}$. L_2_-regularized logistic regression derives the weight vector $\omega$ via solving the optimization problem

4 $$\mathop {min}\limits_{\omega } \frac{1}{2}\omega ^{T} \omega + C\sum\limits_{{i = 1}}^{l} {log\left( {1 + e^{{ - y_{i} \omega ^{T} x_{i} }} } \right)}$$

where $C$ denotes penalty parameter or regularizer. The second term penalizes potential noise/outlier or overtraining. The optimization problem (4) is solved via its dual form

5 $$\begin{aligned} & \mathop {min}\limits_{\alpha } \frac{1}{2}\alpha^{T} Q\alpha + \sum\limits_{{i:\alpha_{i} > 0}}^{l} {\alpha_{i} log\alpha_{i} } + \sum\limits_{{i:\alpha_{i} < C}} {(C - \alpha_{i} )log(C - \alpha_{i} )} - \sum\limits_{i}^{l} {ClogC} \\ & s.t. 0 \le \alpha_{i} \le C,i = 1, \ldots ,l \\ \end{aligned}$$

where ${\alpha }_{i}$ denotes Lagrangian operator and ${Q}_{ij}={y}_{i}{y}_{j}{x}_{i}^{T}{x}_{j}$. To simplify the parameter tuning, the regularizer *C* as defined in Formula (4) is chosen within the set $\{{2}^{i}|-16\le i\le 16,i\in I\}$, where *I* denotes the integer set.

### Metrics for model performance and intensity of drug–drug interactions

#### Metrics for binary classification

Frequently-used performance metrics for supervised classification include Receiver Operating Characteristic curve AUC (ROC-AUC), sensitivity (SE), precision (PR), Matthews correlation coefficient (MCC), accuracy and F1 score. Except that ROC-AUC is calculated based on the outputs of decision function $f(x)$, all the other metrics are calculated via confusion matrix *M.* The element ${M}_{i,j}$ records the counts that class *i* are classified to class *j*. From *M*, we first define several intermediate variables as Formula (6). Then we further define the performance metrics PR_l_, SE_l_ and MCC_l_ for each class label as Formula (7). The overall accuracy and MCC are defined by Formula (8).

6 $$\begin{aligned} & p_{l} = M_{l,l} ,q_{l} = \sum\limits_{i = 1,i \ne l}^{L} {\sum\limits_{j = 1,j \ne l}^{L} {M_{i,j} ,r_{l} } } = \sum\limits_{i = 1,i \ne l}^{L} {M_{i,l} ,s_{l} } = \sum\limits_{j = 1,j \ne l}^{L} {M_{l,j} } \\ & p = \sum\limits_{l = 1}^{L} {p_{l} ,q} = \sum\limits_{l = 1}^{L} {q_{l} ,r} = \sum\limits_{l = 1}^{L} {r_{l} ,s} = \sum\limits_{l = 1}^{L} {s_{l} } \\ \end{aligned}$$

7 $$\begin{aligned} & PR_{l} = \frac{{p_{l} }}{{p_{l} + r_{l} }},l = 1,2 \ldots ,L \\ & SE_{l} = \frac{{p_{l} }}{{p_{l} + s_{l} }},l = 1,2 \ldots ,L \\ & MCC_{l} = \frac{{\left( {p_{l} q_{l} - r_{l} s_{l} } \right)}}{{\sqrt {\left( {p_{l} + r_{l} } \right)\left( {p_{l} + s_{l} } \right)\left( {q_{l} + r_{l} } \right)\left( {q_{l} + s_{l} } \right)} }},l = 1,2 \ldots ,L \\ \end{aligned}$$

8 $$\begin{aligned} & Acc = \frac{{\sum\nolimits_{l = 1}^{L} {M_{l,l} } }}{{\sum\nolimits_{i = 1}^{L} {\sum\nolimits_{j = 1}^{L} {M_{i,j} } } }} \\ & MCC = \frac{{\left( {pq - rs} \right)}}{{\sqrt {\left( {p + r} \right)\left( {p + s} \right)\left( {q + r} \right)\left( {q + s} \right)} }} \\ \end{aligned}$$

where *L* denotes the number of labels and equals to 2 in this study. F1 score is defined as follows.

9 $$F1\;score = \frac{{2 \times PR_{l} \times SE_{l} }}{{PR_{l} + SE_{l} }},\;l = 1\;denotes\;the\;positive\;class$$

#### Metrics for intensity of drug–drug interactions

Two drugs perturbate each other’s efficacy through their targeted genes and the association between the targeted genes determines the interaction intensity of two drugs. If two drugs target common genes or different genes connected via short paths in PPI networks, we deem it as close interaction; if two drugs target different genes via long paths in PPI networks or across signaling pathways, we deem it as distant interaction; otherwise, the two drugs may not interact. If two drugs target common genes, the interaction could be regarded as most intensive and the intensity can be measured by Jaccard index. Given a drug pair (${d}_{i},{d}_{j}$), the Jaccard index between the two drugs is defined as follows

10 $$Jaccard({d}_{i},{d}_{j})=\frac{|{ G}_{{d}_{i}}\cap { G}_{{d}_{j}}|}{|{ G}_{{d}_{i}}\cup { G}_{{d}_{j}}|}$$

where ${G}_{{d}_{i}}$ and ${G}_{{d}_{j}}$ denote the target gene set of ${d}_{i}$ and ${d}_{j}$, respectively. The larger the Jaccard index is, the more intensively the drugs interact. We use the threshold $\xi$ to measure the level of interaction intensity. We further estimate the percentage of drug pairs whose interaction intensity exceeds $\xi$ as follows

11 $${Sim}_{U}=\frac{|\{({d}_{i},{d}_{j})|Jaccard({d}_{i},{d}_{j})\ge \xi ,({d}_{i},{d}_{j})\in U\}|}{|U|}$$

where $U$ denotes the set of drug–drug interactions. If $\xi ={ min}_{\forall ({d}_{i},{d}_{j})\in U}\frac{1}{|{ G}_{{d}_{i}}\cup { G}_{{d}_{j}}|}$, then ${Sim}_{U}$ gives the percentage of drug pairs that target at least one common gene.

Two drugs may also interact through their target genes communicating via protein–protein interactions, although they do not target common genes. In these cases, we need to consider all the paths between two target genes in PPI networks. Given a gene pair (${g}_{i},{g}_{j}$), we use breadth-first graph search algorithm to search for all the paths between $\mathrm{them}$ in human PPI networks, denotes as ${P}_{({g}_{i},{g}_{j})}$. The length of the shortest path and longest path s denoted as ${S}_{({g}_{i},{g}_{j})}$ and ${L}_{({g}_{i},{g}_{j})}$, respectively. We use the distance between target genes in terms of path length in PPI networks to define the distance between drugs. The average number of paths ${Avg}_{({d}_{i},{d}_{j})}$, the shortest distance ${S}_{({d}_{i},{d}_{j})}$ and the longest distance ${L}_{({d}_{i},{d}_{j})}$ between drug ${d}_{i}$ and ${d}_{j}$ are defined as follows.

12 $$\begin{aligned} & Avg_{{\left( {d_{i} ,d_{j} } \right)}} = \frac{{\mathop \sum \nolimits_{{\left( {g_{i} ,g_{j} } \right),g_{i} \in G_{{d_{i} }} \Lambda g_{j} \in G_{{d_{j} }} }} \left| { P_{{\left( {g_{i} ,g_{j} } \right)}} } \right|}}{{\left| {\left\{ {\left( {g_{i} ,g_{j} } \right)\left| {g_{i} \in G_{{d_{i} }} \Lambda g_{j} \in G_{{d_{j} }} } \right.} \right\}} \right|}} \\ & S_{{\left( {d_{i} ,d_{j} } \right)}} = min_{{\forall \left( {g_{i} ,g_{j} } \right),g_{i} \in G_{{d_{i} }} \Lambda g_{j} \in G_{{d_{j} }} }} \;S_{{\left( {g_{i} ,g_{j} } \right)}} \\ & L_{{\left( {d_{i} ,d_{j} } \right)}} = max_{{\forall \left( {g_{i} ,g_{j} } \right),g_{i} \in G_{{d_{i} }} \Lambda g_{j} \in G_{{d_{j} }} }} \;L_{{\left( {g_{i} ,g_{j} } \right)}} \\ \end{aligned}$$

${Avg}_{({d}_{i},{d}_{j})}$ indicates the number of paths through which two drugs interact. ${S}_{({d}_{i},{d}_{j})}$ indicates the most economical and effective way that two drugs interact. ${L}_{({d}_{i},{d}_{j})}$ indicates how far two drugs could alter each other’s efficacy, i.e., action range between two drugs. These three metrics are proposed to measure the interaction intensities between two drugs. Especially, ${S}_{({d}_{i},{d}_{j})}=0$ indicates that drug ${d}_{i}$ and ${d}_{j}$ target common genes, and ${Avg}_{({d}_{i},{d}_{j})}=0$ indicates that there are no paths between drug ${d}_{i}$ and ${d}_{j}$ and the two drugs do not interact.

Assuming *K* signaling pathways in total, if there exists a target gene ${g}_{j}$ of drug ${d}_{i}$ located in a signaling pathway ${Sig}_{k}$, denoted as ${{g}_{j}\in Sig}_{k}$, the pathway set associated with ${g}_{j}$ is defined as ${Sig}_{{g}_{j}}=\{{{{Sig}_{k}|g}_{j}\in Sig}_{k},k=\mathrm{1,2},\dots ,K\}$. The signaling pathways targeted by ${d}_{i}$ is defined as ${\bigcup }_{{g}_{j}\in { G}_{{d}_{i}}}{Sig}_{{g}_{j}}$, and then the common target signaling pathways between ${d}_{i}$ and ${d}_{j}$ are defined as ${Sig}_{({d}_{i},{d}_{j})}={\bigcup }_{{g}_{j}\in { G}_{{d}_{i}}}{Sig}_{{g}_{j}}\bigwedge {\bigcup }_{{g}_{j}\in { G}_{{d}_{j}}}{Sig}_{{g}_{j}}$. The common target cellular processes between ${d}_{i}$ and ${d}_{j}$ are constructed in the same way, except that the signaling pathways are replaced with the GO terms of biological processes in GOA database^39^.

## Results

### Performance of cross validation and independent test

**Figure 1:**
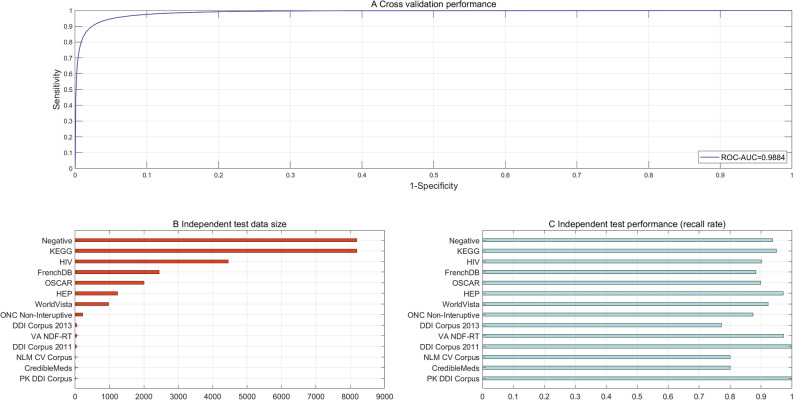
Performance of cross validation and independent test. (**A**) ROC curve and AUC score for fivefold cross validation. (**B**) Statistics of independent test data size. (**C**) Recall rates on the independent test data.

The results of fivefold cross validation show that the proposed framework fairly encouraging performance (see Fig. 1A for ROC-AUC scores and Table 1 for other metrics). The metrics of SP, SE and MCC on the two classes show that the proposed framework is less biased, e.g., 0.9556 on the positive class, 0.9402 on the negative class in terms of sensitivity and 0.9007 overall MMC. These results show that drug target profile alone is sufficient to separate interacting drug pairs from non-interacting drug pairs with a high accuracy (Accuracy = 94.79%). Drug takes effect via its targeted genes and the direct or indirect association or signaling between targeted genes underlies the mechanism of drug–drug interaction. From this aspect, drug target profile intuitively and effectively elucidates the molecular mechanism behind drug–drug interactions. Drug target profile could represent not only the genes targeted by structurally similar drugs but also the genes targeted by structurally dissimilar drugs, so that it is less biased than drug structural profile. The results also show that neither data integration nor drug structural information is indispensable for drug–drug interaction prediction. To more objectively gain knowledge about whether or not the model behaves stably, we evaluate the model performance with varying *k*-fold cross validation (*k* = 3, 5, 7, 10, 15, 20, 25) (see the Supplementary Fig. S1). The results show that the proposed framework achieves nearly constant performance in terms of Accuracy, MCC and ROC-AUC score with varying *k*-fold cross validation.

**Table 1:** Performance estimation of fivefold cross validation and independent test.

Cross validation							Independent test (recall rate)			
PR	SE	MCC	Acc	MCC*	AUC	F1 score	KEGG	OSCAR	VA NDF-RT	Negative
0.9411 (+) 0.9549 (−)	0.9556 (+) 0.9402 (−)	0.9009 (+) 0.9007 (−)	94.79%	0.9007	0.9884	0.9483	0.9497	0.8992	0.9730	0.9373

The bracketed + denotes positive class, the bracketed − denotes negative class and MCC* denotes overall MCC.

Cross validation still is prone to overfitting, though that the validation set is disjoint with the training set for each fold. We further conduct independent test on 13 external DDI datasets and one negative independent test data to estimate how well the proposed framework generalizes to unseen examples. The size of the independent test data varies from 3 to 8188 (see Fig. 1B). The performance of independent test is in Fig. 1C. The proposed framework achieves recall rates on the independent test data all above 0.8 except the dataset “DDI Corpus 2013”. On the experimental DDIs from KEGG^26^, OSCAR^27^ and VA NDF-RT^28^, the proposed framework achieves recall rate 0.9497, 0.8992 and 0.9730, respectively (see Table 1). On the negative independent test data, the proposed framework also achieves 0.9373 recall rate, which indicates a low risk of predictive bias. The independent test performance also shows that the proposed framework trained using drug target profile generalizes well to unseen drug–drug interactions with less bias.

### Comparisons with existing methods

Existing methods infer drug–drug interactions majorly via drug structural similarities in combination with data integration in many cases. Structurally similar drugs tend to target common or associated genes so that they interact to alter each other’s therapeutic efficacy. These methods surely capture a fraction of drug–drug interactions. However, structurally dissimilar drugs may also interact through their targeted genes, which cannot be captured by the existing methods based on drug structural similarities. In our proposed framework, direct or indirect associations between the target genes of two drugs are assumed to be the major driving force that induces drug–drug interactions, so as to capture both structurally-similar and structurally-dissimilar drug–drug interactions. From biological insights, the proposed framework is easier to interpret. From computational point of view, the proposed framework uses drug target profiles only and greatly reduces data complexity as compared to existing data integration methods.

From performance point of view, the proposed framework also outperforms existing methods. The performance comparisons are provided in Table 2. All the existing methods achieve fairly high ROC-AUC scores except Cheng et al.^15^ (ROC-AUC = 0.67). Unfortunately, these methods show a high risk of bias. For instance, the model proposed by Vilar et al.^9^, trained via drug structural profiles, is highly biased towards the negative class with sensitivity 0.68 and 0.96 on the positive and the negative class, respectively. The data integration method proposed by Zhang et al.^19^ achieves encouraging performance of cross validation (ROC-AUC score = 0.957, PR = 0.785, SE = 0.670) but only recognizes 7 out of 20 predicted DDIs (equivalent to 35% recall rate of independent test), although it exploits a large amount of feature information such as drug substructures, drug targets, drug enzymes, drug transporters, drug pathways, drug indications and drug side-effects. Similarly, Gottlieb et al.^23^ achieve fairly good performance of cross validation but achieve only 53% recall rate of independent test.

**Table 2:** Performance comparisons with existing methods.

	Cross validation					Independent test	
	PR	SE	MCC	F1 score	ROC-AUC		
Vilar et al.^7^	0.26 (+) 11.81 (−)	0.68 (+) 0.96 (−)	–	–	0.92		31%
Ferdousi et al.^8^	–	0.72 (+)	–	–	–		–
Cheng et al.^16^	–	–	–	–	0.67		–
Zhang et al.^17^	0.785	0.670	–	0.723	0.957		35%
Song et al.^18^	0.68 (+)	–	–	–	0.9738		24%
Gottlieb et al.^21^	0.88	0.93	–	–	0.96		53%
Karim et al.^23^	–	–	0.79	0.91	0.97		–

The bracketed sign + denotes positive class, the bracketed sign − denotes negative class and the other sign – denotes missing values.

Deep learning, the most promising revolutionary technique to date in machine learning and artificial intelligence, has been used to predict the effects and types of drug–drug interactions^21,22^. The most related deep learning framework proposed by Karim et al.^25^ automatically learns feature representations from the structures of available drug–drug interaction networks to predict novel DDIs. This method also achieves satisfactory performance (ROC-AUC score = 0.97, MCC = 0.79, F1 score = 0.91), but the learned features are hard to interpret and to provide biological insights into the molecular mechanisms underlying drug–drug interactions.

### Analyses of molecular mechanisms behind drug–drug interactions

#### Jaccard index between two drugs

**Figure 2:**
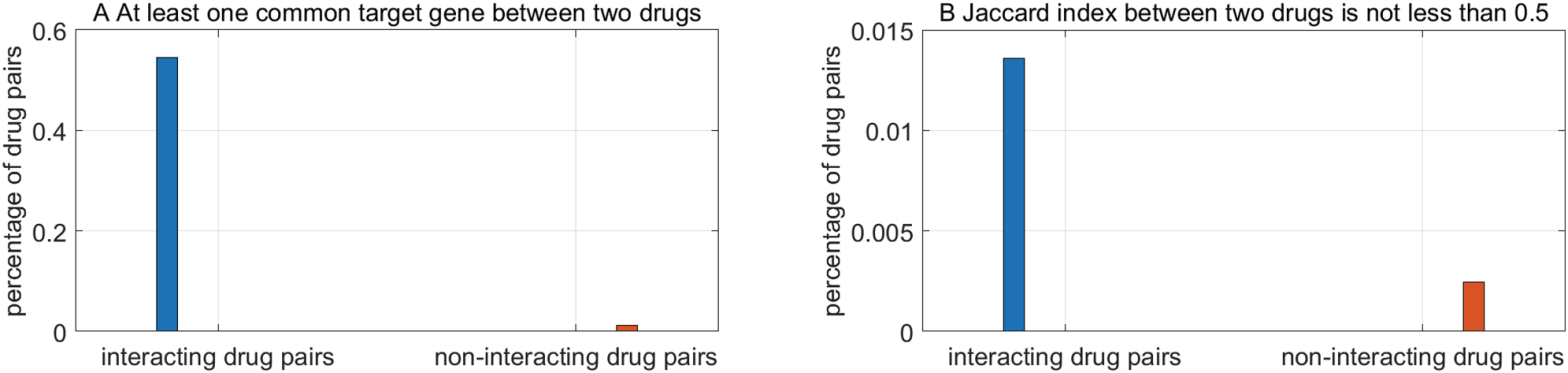
Statistics of common target genes between interacting and non-interacting drugs.

The more common genes two drugs target, the more intensively the two drugs potentially interact. As presented in Formula (10), the interaction intensity is measured with Jaccard index. The percentage of drug pairs whose interaction intensity exceeds ξ is illustrated in Fig. 2. The threshold of interaction intensity assumes $\xi ={ min}_{\forall ({d}_{i},{d}_{j})\in U}\frac{1}{|{ G}_{{d}_{i}}\cup { G}_{{d}_{j}}|}$ and $\xi =0.5$ in Fig. 2A,B, respectively. The statistics are derived from the training data. We can see that interacting drugs tend to target much more common genes than non-interacting drugs.

#### Average number of paths between two drugs

**Figure 3:**
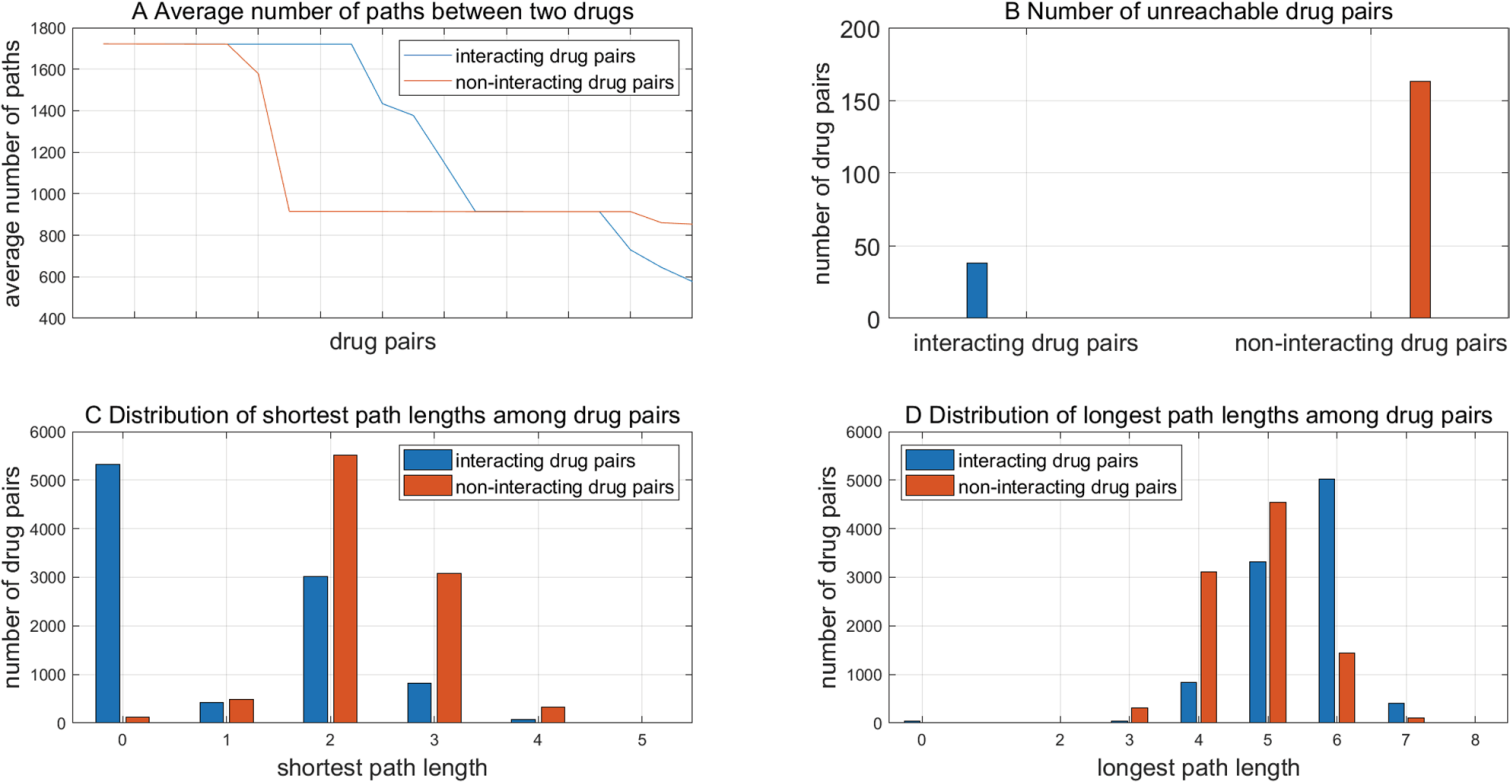
The statistics of average number of paths, shortest path lengths and longest path lengths between two drugs.

The average number of paths between the garget genes of two drugs as defined in Formula (12) also measures the interaction intensity between drugs. To reduce the time of paths search, we only randomly choose 9692 interacting drug pairs and 9692 non-interacting drug pairs as examples for the analyses of molecular mechanism behind drug–drug interactions. The average number of paths of top twenty drug pairs are illustrated in Fig. 3A. We can see that interacting drug pairs have their target genes more heavily connected than non-interacting drug pairs, which also means the more paths two drugs are connected through, the more probably the two drugs interact to alter each other’s effects. As shown in Fig. 3B, non-interacting drugs are more likely to be unreachable to each other than interacting drugs.

#### Shortest path length between two drugs

For the randomly sampled 9692 interacting drug pairs and 9692 non-interacting drug pairs, the length of the shortest paths between two drugs’ target genes ranges from 0 to 5 (see Fig. 3C). We can see that interacting drug pairs significantly outnumber non-interacting drug pairs when the shortest path length is equal to 0, that’s, that two drugs target common genes. With the increase of the shortest path length, non-interacting drug pairs gradually outnumber interacting drug pairs. These results show that drug–drug interactions tend to occur between drugs that target common genes or whose target genes come across via shorter shortest paths. The shorter the shortest path is, the more efficiently the drugs interact.

#### Longest path length between two drugs

For the randomly sampled drug pairs, the length of the longest paths between two drugs’ target genes ranges from 0 to 8 (see Fig. 3D). Non-interacting drug pairs outnumber interacting drug pairs when the longest path ranges from 3 to 5, but conversely interacting drug pairs significantly outnumber non-interacting drug pairs when the longest path length equals to 6. These results to some extent show that interacting drugs could exert far-reaching perturbations on each other with a longer range of action than non-interacting drugs. The metrics ${Avg}_{({d}_{i},{d}_{j})},$ ${S}_{({d}_{i},{d}_{j})}$ and ${L}_{({d}_{i},{d}_{j})}$ defined in Formula (12) could measure the tendency of drug–drug interaction in terms of interaction intensity, interaction efficiency and action range. When the shortest path length equals to 0 and the longest path length equals to 6, the randomly sampled interacting and on-interacting drug pairs show a significant statistical difference.

#### Common target pathways between two drugs

**Figure 4:**
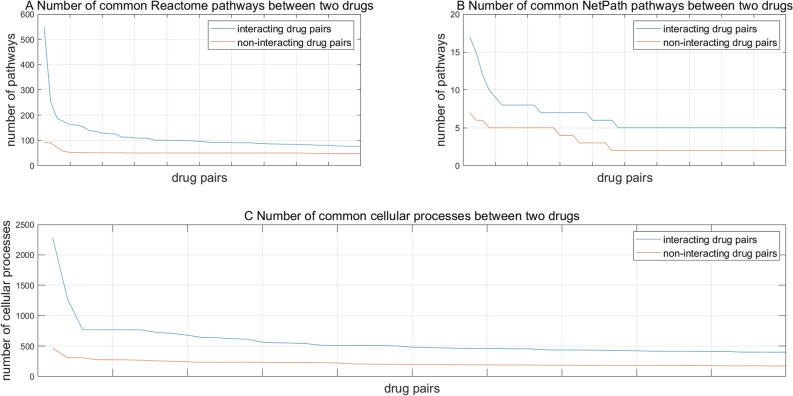
Statistics of common signaling pathways that two drugs target and common cellular processes that two drugs are involved in.

We map the target genes onto the signaling pathways from NetPath^36^ and Reactome^37^ to investigate that interacting drugs tend to target common signaling pathways. Computational results show that interacting drug pairs tend to target more common signaling pathways than non- interacting drug pairs (see Fig. 4A for NetPath pathways and Fig. 4B for Reactome pathways). If the target genes of two drugs are located in the same signaling pathway, the two drugs are more likely to perturbate each other’s efficacies.

#### Common cellular processes between two drugs

As shown in Fig. 4C, interacting drugs are more likely to get involved in common cellular processes than non-interacting drugs. This phenomenon is not hard to understand. Two drugs whose target genes are involved in common cellular processes more likely alter each other’s therapeutic effects.

### Predictions and clinical implications

We randomly sample 99,986 drug pairs as the prediction set, which are not overlapped with the training data and the independent test data. Thereinto, 43,719 drug pairs are predicted to interact by the proposed framework (see Supplementary File S1). These predictions to some extent contain a certain level of false interactions. For each prediction, a confidence level in the form of probability could be chosen to filter out the weak interactions (e.g., 0.7 probability as a threshold). These predictions are further analysed from the aspect of cellular processes (see Supplementary File S2) and signaling pathways (see Supplementary File S3) to help us understand the molecular mechanisms underlying drug–drug interactions. We choose the drug Nabiximols and Glucosamine as a case study.

Nabiximols (C_42_H_60_O_4_), extracted from *Cannabis sativa L.,* is often used to treat neuropathic pain and intractable cancer pain, with the pharmacological effects of analgesic, muscle relaxant, anxiolytic, neuroprotective and anti-psychotic activity (https://www.drugbank.ca/drugs/DB14011). Glucosamine (C_6_H_13_NO_5_), as a precursor for glycosaminoglycans that are a major component of joint cartilage, is often used to rebuild cartilage and treat osteoarthritis (https://www.drugbank.ca/drugs/DB01296). According to DrugBank^27^, Nabiximols targets 57 human genes and Glucosamine targets six human genes. Based on these target genes, we could analyse the cellular processes and signaling pathways through which Nabiximols and Glucosamine take effect.

#### Common cellular processes between Nabiximols and Glucosamine

**Figure 5:**
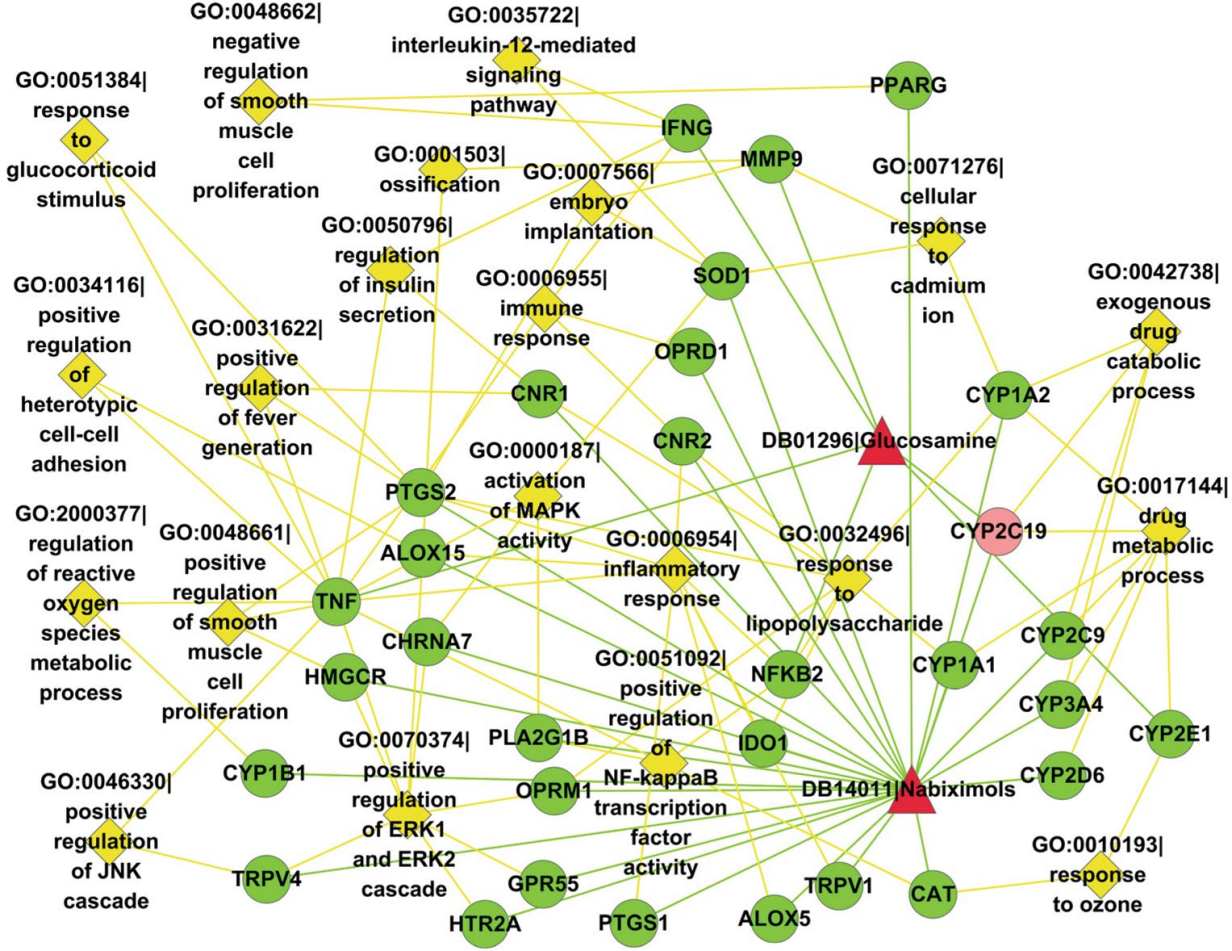
Common cellular processes of target genes between DB14011|Nabiximols and DB01296|Glucosamine predicted to interact. Red triangle nodes denote drugs; green circle nodes denote drug target genes; light red circle nodes denote common target genes; and yellow diamond nodes denote biological processes of gene ontology. This drawing is created by Cytoscape version 2.8.2 (https://cytoscape.org/).

Two drugs mediate common cellular processes via common target genes or associated target genes involved in the same cellular processes. Computational results show that Nabiximols and Glucosamine get involved 68 common cellular processes. For clarity, only 21 cellular processes and the associated target genes are illustrated in Fig. 5. The rest cellular processes are provided in Supplementary File S2. As shown in Fig. 5, Nabiximols and Glucosamine mediate the common cellular processes of exogenous drug catabolic process (GO:0042738) and drug metabolic process (GO:0017144) via the common gene *CYP2C19*. Association via different target genes is one major way that two drugs mediate common cellular processes. For instance, Nabiximols and Glucosamine mediate the common cellular processes of negative regulation of smooth muscle cell proliferation (GO:0048662) via Nabiximols-targeted gene *PPARG* and Glucosamine-targeted gene *IFNG*. For another example, Nabiximols and Glucosamine mediate the common cellular processes of regulation of reactive oxygen species (ROS) metabolic process (GO:2000377) via Nabiximols-targeted gene *CYP1B1* and Glucosamine-targeted gene *TNF*. Among the predicted drug–drug interactions, many drug pairs do not target common genes but they are found to mediate common cellular processes via different target genes (see Supplementary File S2). For instance, drug Nabiximols (DB14011) and Gallium nitrate (DB05260) are not found to target common genes in DrugBank^27^, but they are predicted to target the common cellular processes of neutrophil chemotaxis (GO:0030593), positive regulation of NF-kappaB transcription factor activity (GO:0051092), etc.

#### Common signaling pathways between Nabiximols and Glucosamine

**Figure 6:**
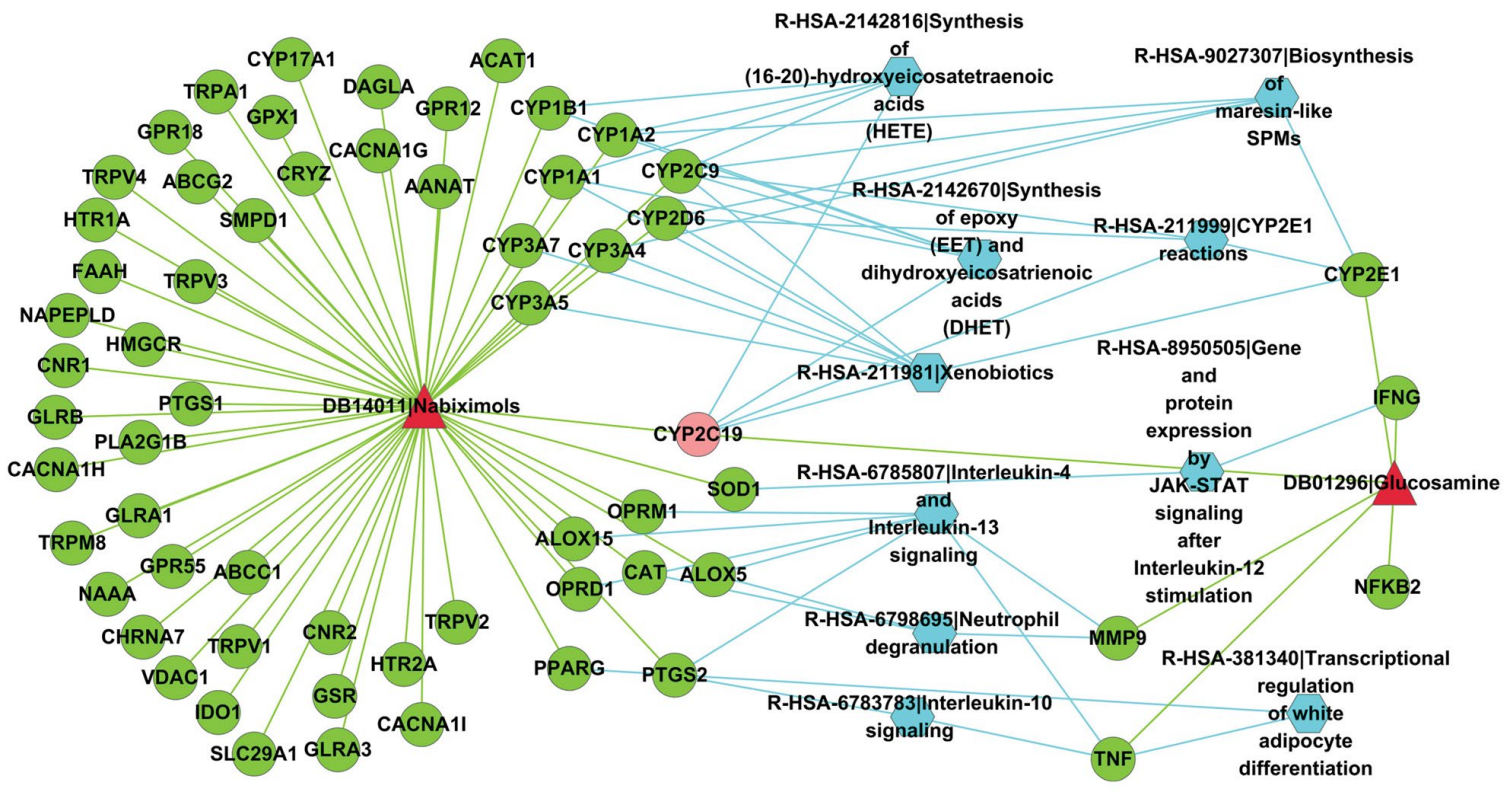
Common target Reactome signaling pathways between DB14011|Nabiximols and DB01296|Glucosamine predicted to interact. Red triangle nodes denote drugs; green circle nodes denote drug target genes; light red circle nodes denote common target genes; and blue hexagon nodes denote Reactome signaling pathways. This drawing is created by Cytoscape version 2.8.2 (https://cytoscape.org/).

The common Reactome signaling pathways that Nabiximols and Glucosamine mediate are illustrated in Fig. 6. Among the target genes, the common target gene *CYP2C19* is located in four Reactome signaling pathways, i.e., Synthesis of epoxy (EET) and dihydroxyeicosatrienoic acids (DHET) (R-HSA-2142670), Xenobiotics (R-HSA-211981), CYP2E1 reactions (R-HSA-211999) and Synthesis of (16-20)-hydroxyeicosatetraenoic acids (HETE) (R-HSA-2142816). Apart from common garget genes, association via different target genes also leads to two drugs mediating common signaling pathways. For instance, Nabiximols and Glucosamine mediate the common signaling pathway of Neutrophil degranulation (R-HSA-6798695) via Nabiximols-targeted gene *ALOX5* and Glucosamine-targeted gene *MMP9*. Two drugs that do not target common genes also potentially mediate the same signaling pathways (see Supplementary File S3). For instance, drug Nabiximols (DB14011) and SF1126 (DB05210) have not been reported to target common genes in DrugBank^27^, but they are predicted to mediate several common signaling pathways, e.g., Regulation of PTEN gene transcription (R-HSA-8943724), Interleukin-4 and Interleukin-13 signaling (R-HSA-6785807), G alpha (q) signaling events (R-HSA-416476).

## Discussion

Only after co-prescribed drugs have clinically done damages to patient health and life, could drug–drug interactions be detected and reported in most cases. For this reason, we need resort to computational methods to predict whether two drugs interact and produce undesirable side effects before clinical co-prescription. Existing computational methods focus on integrating multiple heterogeneous data sources to increase model performance, among which drug structural profile is the most frequently used feature information. These methods heavily depend on drug structures and assume that structurally similar drugs often target common or associated genes so as to alter each other’s therapeutic efficacies. This assumption surely captures a fraction of drug–drug interactions but shows bias, because it ignores a large fraction of interactions between structurally dissimilar drugs. The other major drawback of these methods lies in the high data complexity. In these methods, we do not know which information contributes most to the model performance and it is hard to interpret the molecular mechanisms behind drug–drug interactions. Furthermore, data integration would fail when the required data are not available, e.g., drug structures, drug side-effects, clinical records. Lastly, proper representation of drug molecule structures and extracting features from drug SMILES remain challenging in the progress of computational modelling for drug development. In this study, we use drug target profile to depict drugs and drug pairs to achieve two goals. One goal is to simplify the modeling processes via reducing data complexity and relieving dependency on drug molecular structures. The other goal is to computationally model the molecular mechanisms underlying drug–drug interactions so that the model is biologically interpretable. Drugs act on their target genes to produce desirable therapeutic efficacies. We assume that the perturbations of two drugs come across through common target genes, paths in PPI networks or signaling pathways, synergistic enhancement or antagonistic counteract of therapeutic effects of individual drugs would take place. As compared to the existing methods, this proposed framework bases the assumption of drug–drug interactions on drug–targeted genes instead of drug structural similarities. We use the known drug–drug interactions from DrugBank^27^ as the positive training data and randomly sample the same size of drug pairs as the negative training data to train an l_2_-regualrized logistic regression model. *K*-fold cross validation is a common practice used to estimate model performance, but the performance varies with the choice of *k*. The best practice is to choose *k* at intervals (e.g., *k* = 3, 5, 10, 15, …) or even conduct leave-one-out cross validation, so that we could more objectively know whether or not the model behaves stably. However, this practice is computationally prohibitive to large training data (915,413 positive examples and 915,413 negative examples) and thirteen external test datasets with tedious model parameters tuning. Actually, it is hard to obtain a training set representative of and infinitely approximate to the population distribution via varying *k*-folds. Nevertheless, we still evaluate the model performance with varying *k*-fold cross validation (*k* = 3, 5, 7, 10, 15, 20, 25). The results show that the performance in terms of Accuracy, MCC and ROC-AUC score is fairly stable with *k* varying widely. Apart from horizontally randomizing examples (X-randomization), some statistical machine learning models such as Random Forest also conduct vertical feature randomization (Y-randomization) to obtain different views or to evaluate feature importance. Because the known target genes are very sparse and thus random sampling of feature subsets potentially results in null vector representation of drug pairs, we choose all the features in this study.

Empirical studies show that the proposed framework achieves fairly encouraging performance of fivefold cross validation and independent test on thirteen external datasets, which significantly outperforms the existing methods. Furthermore, the encouraging performance on the randomly sampled negative independent test data shows that the proposed framework is less biased. Nevertheless, the proposed framework yields a little large fraction of false interactions, which is largely due to the quality of randomly sampled negative training data. This problem could be to some extent solved by choosing a higher threshold of probability to filter out the weak predictions. In addition, drug target profile simplifies computational modeling, but meanwhile restricts the application of the proposed framework in that the target genes have not been reported for many less-studied drugs. This problem could be solved with the accumulation of the knowledge about drug target genes. The proposed framework could to some extent to be generalized to the other problems concerned with drug discovery, e.g., drug combinatorial synergy and antagonism, drug side-effects, drug–food interaction, etc., in which drug target profile could still be useful. Whether or not drug target profile representation is sufficient to solve these problems need to be further investigated.

We further propose several statistical metrics based on protein–protein interaction networks and signaling pathways to measure the intensity that drugs act on each other. These metrics show that two drugs tend to interact more efficiently if their perturbations could come across via shorter shortest paths in PPI networks, and the perturbations would be more far-reaching if longer shortest paths between the two drugs. Lastly, we use the common cellular processes and signaling pathways that two drugs target to understand the mechanisms underlying drug–drug interactions. The unravelled mechanisms are useful to provide biological insights into potential pharmacological risks of known drug–drug interactions.

## Conclusions

Drug target profile representation of drugs and drug pairs simplifies the modeling processes for drug–drug interactions by reducing both data complexity and dependency on drug molecular structures. Meanwhile, Drug target profile representation renders the proposed framework biologically interpretable in terms of molecular mechanisms underlying drug–drug interactions.

## Supplementary Information

Supplementary Figure S1.

Supplementary Information 1.

Supplementary Information 2.

Supplementary Information 3.

## Data Availability

The source code and tools for this proposed framework are publicly available at https://github.com/suyumei/DrugDrugIntact.git.
