# Supplementary material for: A machine learning framework for predicting drug–drug interactions
Source: Sci Rep. 2021 Sep 2;11:17619. doi: 10.1038/s41598-021-97193-8 (PMC8413337; doi:10.1038/s41598-021-97193-8)
Supplement: Supplementary file 1 — Supplementary Figure S1. [file 41598_2021_97193_MOESM1_ESM.pdf]

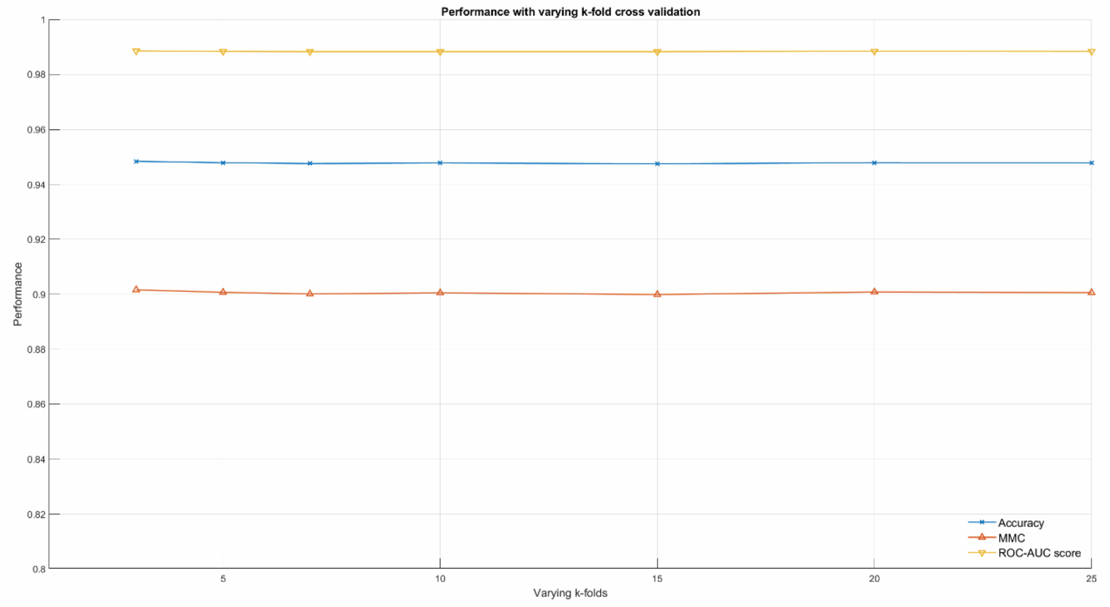

Figure S1 Performance with varying *k*-fold cross validation (*k*=3, 5, 7, 10, 15, 20, 25) in terms of Accuracy, MCC and ROC-AUC score.
